# Crisaborole Topical Ointment, 2% in Patients Ages 2 to 17 Years with Atopic Dermatitis: A Phase 1b, Open‐Label, Maximal‐Use Systemic Exposure Study

**DOI:** 10.1111/pde.12872

**Published:** 2016-05-18

**Authors:** Lee T. Zane, Leon Kircik, Robert Call, Eduardo Tschen, Zoe Diana Draelos, Sanjay Chanda, Merrie Van Syoc, Adelaide A. Hebert

**Affiliations:** ^1^ Anacor PharmaceuticalsPalo AltoCalifornia; ^2^ DermResearchLouisvilleKentucky; ^3^ St. Louis UniversitySt LouisMissouri; ^4^ Academic Dermatology AssociatesAlbuquerqueNew Mexico; ^5^ Dermatology Consulting ServicesHigh PointNorth Carolina; ^6^ University of Texas Health Science CenterHoustonTexas

## Abstract

**Background:**

Phosphodiesterase‐4 (PDE4) is a promising target in atopic dermatitis (AD) treatment. The pharmacokinetics (PK), safety, and efficacy of crisaborole topical ointment, 2% (formerly AN2728) (Anacor Pharmaceuticals, Palo Alto, CA), a boron‐based benzoxaborole PDE4 inhibitor, were evaluated in children with mild to moderate AD.

**Methods:**

This phase 1b, open‐label, maximal‐use study of crisaborole topical ointment, 2% applied twice daily (dose 3 mg/cm^2^) for 28 days enrolled patients ages 2 to 17 years with extensive AD involving 25% or more or 35% or more treatable body surface area, depending on age. Primary PK and safety assessments included systemic exposure to crisaborole and its metabolites after 7 days of treatment and the incidence of treatment‐emergent adverse events (TEAEs). Secondary efficacy assessments included change from baseline in Investigator Static Global Assessment (ISGA), treatment success (ISGA score ≤1 with a two‐grade or greater improvement from baseline), and improvement in five AD signs and symptoms.

**Results:**

Of 34 patients enrolled, 31 completed the study. Crisaborole was rapidly absorbed, with limited systemic exposure between days 1 and 8. Twenty‐three of 34 patients reported one or more TEAEs; 95% were mild or moderate and one patient discontinued because of a TEAE. Mean ISGA scores declined from 2.65 at baseline to 1.15 at day 29, 47.1% of patients achieved treatment success, and 64.7% of patients achieved ISGA scores of clear (0) or almost clear 1. Mean severity scores for AD signs and symptoms declined throughout the study.

**Conclusions:**

This open‐label study provides evidence that crisaborole topical ointment, 2% was well tolerated, with limited systemic exposure under maximal‐use conditions in patients ages 2 years and older.

Atopic dermatitis (AD) is a chronic, relapsing, pruritic inflammatory disease associated with skin barrier dysfunction, intense pruritus, and eczematous skin lesions 1, 2. AD affects an estimated 15% to 30% of children and 2% to 10% of adults in industrialized countries and causes significant difficulties in health‐related quality of life from disease symptoms and the stigma associated with a highly visible skin condition 1, 3. Most patients with AD have mild to moderate disease and are often treated with topical therapies; systemic modalities are recommended only for patients with moderate to severe disease 4. Patients with mild to moderate AD are typically treated with topical emollients, corticosteroids, and calcineurin inhibitors 2. These agents are effective in many cases, but topical corticosteroids and calcineurin inhibitors have the potential to induce unwanted side effects, which may be of particular concern for caregivers of young children 2.

The intracellular enzyme phosphodiesterase‐4 (PDE4) has emerged as a promising target in the treatment of inflammatory skin diseases, including AD and psoriasis 5, 6. The inhibition of PDE4 blocks the conversion of the intracellular second messenger cyclic adenosine monophosphate (cAMP) to 5′‐AMP and facilitates interaction between cAMP and protein kinase A to prevent activation of proinflammatory cytokines 5. Systemic therapy with PDE4 inhibitors is associated with gastrointestinal adverse events (especially nausea), so topical therapy appears to be the optimal approach with respect to PDE4 inhibition in AD treatment 6, 7.

Crisaborole (formerly AN2728; Anacor Pharmaceuticals, Palo Alto, CA) is a novel, boron‐based nonsteroidal anti‐inflammatory PDE4 inhibitor that has demonstrated antiinflammatory properties and inhibition of cytokine release in biochemical, cell‐based, and animal studies 8, 9. The presence of boron within the chemical structure of crisaborole is essential for inhibition of PDE4 activity; substitution of boron for carbon eliminates this inhibitory effect. As a small (251 Da) lipophilic compound demonstrating greater skin penetration than that of related compounds studied, in combination with significant anti‐inflammatory activity, crisaborole was selected as the lead development candidate for treatment of AD 8, 9.

The objective of the current open‐label, phase 1b study was to evaluate the systemic exposure, pharmacokinetics (PK), safety, and efficacy of crisaborole topical ointment, 2% under maximal‐use conditions in children and adolescents with mild to moderate AD.

## Methods and Materials

### Study Design

This was a phase 1b, multicenter, open‐label, maximal‐use study of crisaborole topical ointment, 2% applied twice daily (BID). The study included a PK and safety phase (dose application in clinic from day 1 through the morning dose on day 9) and a non‐PK safety phase (at‐home dose application from the evening dose on day 9 through day 28). The study was approved by the Human Research Ethics Committee (HREC), the Bellberry Human Research Ethics Committee (10 sites), and the Alfred Hospital Ethics Committee (4 sites) before initiation and was conducted in accordance with Good Clinical Practice guidelines and the US Code of Federal Regulations and pursuant to the Declaration of Helsinki. All patients (if appropriate) provided assent and their parents or guardians provided informed written consent before enrollment. The primary objective was to evaluate the systemic exposure, PK, and safety of crisaborole topical ointment, 2% when applied under maximal‐use conditions in children and adolescents 2 to 17 years of age. Secondary objectives included efficacy assessments of crisaborole topical ointment, 2%. The inclusion and exclusion criteria are summarized in Table 1.

**Table 1:** Summary of Inclusion and Exclusion Criteria for Study Patients

Inclusion criteria	Exclusion criteria
Boys and girls with a confirmed diagnosis of AD according to the criteria of Hanifin and Rajka 16 Minimum AD involvement for treatable percentage of BSA depending on age Cohort 1: 12–17 yrs; treatable BSA ≥25% Cohort 2: 6–11 yrs: treatable BSA ≥35% Cohort 3: 2–5 yrs; treatable BSA ≥35% Investigator Static Global Assessment score of 2 or 3 at baseline Normal clinical laboratory results Adequate unaffected skin area to permit repeat venous sampling for pharmacokinetic analysis	Clinically significant physical or mental disorder Unstable AD or a consistent requirement for high‐potency^a^ corticosteroids Active systemic or localized infection (including infected AD) History or evidence of clinically significant or severe allergies Using systemic or topical therapies that might alter the course of AD, including: High‐ or mid‐potency topical corticosteroids or calcineurin inhibitors within 2 wks of baseline Topical hydrocortisone 1% within 3 days of baseline Topical antihistamines within 1 wk of baseline Systemic corticosteroids or immunosuppressive agents within 4 wks of baseline Systemic antiinflammatory or immunomodulatory drugs (biologics) within 2 wks of baseline Systemic antihistamines were allowed only if regimen was not modified during study^b^ Use of systemic antibiotics within 2 wks of baseline

AD, atopic dermatitis; BSA, body surface area.

a Patients demonstrating a consistent requirement for class I–III steroids were excluded.

b Patients following stable regimens (≥2 wks consistent use before study baseline) with systemic antihistamines, dilute bleach baths, topical retinoid, or topical benzoyl peroxide were permitted to continue use but could not alter or stop the regimen during the study.

### Study Treatments

Crisaborole topical ointment, 2% was applied BID to all treatable AD‐affected areas at an intended dose of 3 mg/cm^2^. The per‐application dosage was calculated in grams at baseline for each patient based on total body surface area (BSA) and treatable percentage of BSA (percentage of AD‐involved skin excluding scalp and venous access areas). During the PK phase, the per‐application dosage remained fixed at the baseline level and was applied BID, with the evening application occurring 8 to 12 hours after the morning application. (On days 1 and 8, the study drug was applied only in the morning.) During the non‐PK safety phase, patients or caregivers administered per‐application doses to all AD‐involved skin identified at baseline (regardless of whether areas had become clinically clear) using the same timing and application guidelines employed in the PK phase; additional study drug could be administered to new AD lesions at the discretion of study investigators.

### Study Assessments

#### PK and Safety

The primary study endpoints were PK and safety of crisaborole topical ointment, 2% under maximal‐use conditions. PK profiles were developed for crisaborole and its major oxidative metabolites AN7602 and AN8323 based on plasma samples obtained on days 1 and 8. Calculated PK parameters included area under the curve (AUC), maximum plasma concentration (*C*_max_), time to *C*_max_ (*T*_max_), and terminal half‐life (*t*_1/2_), assessed on days 1 and 8.

The safety of crisaborole topical ointment, 2% was assessed using tabulation of treatment‐emergent adverse events (TEAEs) and serious adverse events (SAEs), which were assessed at baseline and days 2 through 9, 15, 22, and 29 (posttreatment follow‐up). A TEAE was defined as any adverse event that developed or worsened after the first dose of study medication through study completion or termination. An SAE was defined as any adverse event that was life threatening, required hospitalization, or resulted in significant disability or death. Vital signs were assessed at baseline and days 2, 5, 8, 9, 15, 22, and 29; blood samples for laboratory assessments were obtained at baseline and days 8 and 29. A full physical examination was conducted at screening and a disease‐focused physical examination was conducted at baseline and days 8, 15, 22, and 29.

#### Efficacy

Secondary assessments included treatment success, defined as an Investigator Static Global Assessment (ISGA) score of 1 or less (clear or almost clear) with a two‐grade improvement or more from baseline. The proportion of patients achieving an ISGA of 0 (clear) or 1 (almost clear) was also assessed. Improvement from baseline in AD signs and symptoms of erythema, excoriation, exudation, lichenification, and pruritus was evaluated using a 4‐point scale ranging from 0 (none) to 3 (severe). Efficacy assessments were conducted at baseline and days 5, 8, 15, 22, and 29. Change from baseline in treatable percentage of BSA was assessed at day 29 and included all AD lesions identified at baseline.

### Statistical Analysis

Plasma PK samples were evaluated for crisaborole, AN7602, and AN8323 using a validated high‐performance liquid chromatography tandem mass spectrometry assay and analyzed using analysis of variance (ANOVA). Descriptive PK parameters were determined using standard noncompartmental methods based on concentration‐versus‐time curves for each patient.

**Figure 1:**
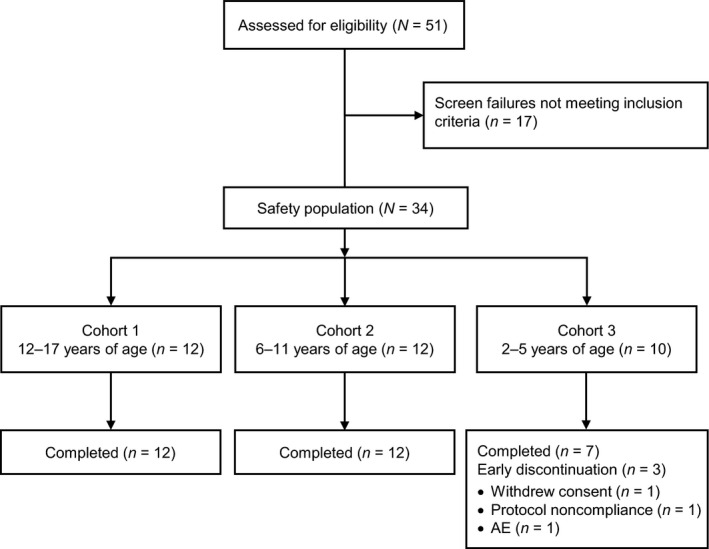
Patient disposition. AE, adverse event.

Efficacy and safety analyses were conducted using SAS version 9.1 or higher (SAS Institute, Cary, NC) using descriptive statistics. Continuous variables were described using number, mean, standard deviation (SD), median, minimum, and maximum; categorical variables were summarized using counts and percentages. This study was not designed with the power or sample size to evaluate statistical significance, and inferential statistics were not reported, but an ANOVA model was used to compare *C*_max_ and AUC from time 0 to 24‐hour postdosing (AUC_0–24_) values for AN2728, AN7602, and AN8323 in the three patient cohorts (see Fig. 1), with statistical significance set at p ≤ 0.05. If no statistically significant differences were observed using this model, the PK analysis was performed for all study patients.

## Results

### Study Population

Thirty‐four patients (*n* = 12 in cohort 1, *n* = 12 in cohort 2, and *n* = 10 in cohort 3) were enrolled and received treatment with crisaborole topical ointment, 2% (safety population). Thirty‐one patients completed the study: all 24 patients in cohorts 1 and 2 and 7 in cohort 3 (Fig. 1). Reasons for discontinuation in cohort 3 were withdrawal of consent or reasons unrelated to study treatment (*n* = 1), nonadherence to protocol (*n* = 1), and a TEAE (*n* = 1; application site pain). Patient demographic and baseline clinical characteristics are summarized in Table 2.

**Table 2:** Summary of Demographic and Baseline Clinical Characteristics of Study Patients: Safety Population^a^

Characteristic	Cohort 1, ages 12–17 yrs (*n* = 12)	Cohort 2, ages 6–11 yrs (*n* = 12)	Cohort 3, ages 2–5 yrs (*n* = 10)	Total (*N* = 34)
Sex, *n* (%)				
Male	7 (58)	4 (33)	4 (40)	15 (44)
Female	5 (42)	8 (67)	6 (60)	19 (56)
Age, yrs, mean ± SD	14.3 ± 1.85	8.9 ± 1.41	3.7 ± 1.10	9.3 ± 4.54
Race, *n* (%)				
White	6 (50)	5 (42)	6 (60)	17 (50)
Black	5 (42)	6 (50)	4 (40)	15 (44)
Native Hawaiian or Pacific Islander	1 (8)	1 (8)	0	2 (6)
Height, cm, mean ± SD	164.8 ± 9.7	131.7 ± 10.3	97.5 ± 8.8	133.3 ± 29.0
Weight, kg, mean ± SD	65.0 ± 18.4	33.3 ± 11.7	16.5 ± 3.78	39.6 ± 23.9
Investigator Static Global Assessment disease severity score, mean ± SD	2.67 ± 0.49	2.58 ± 0.52	2.70 ± 0.48	2.65 ± 0.49
Treatable percentage of body surface area, mean ± SD	35.8 ± 11.3	54.9 ± 19.3	56.9 ± 23.0	48.7 ± 20.2

SD, standard deviation.

a The safety population included all enrolled patients who received at least one dose of study medication and at least one postbaseline assessment.

### PK Results

**Figure 2:**
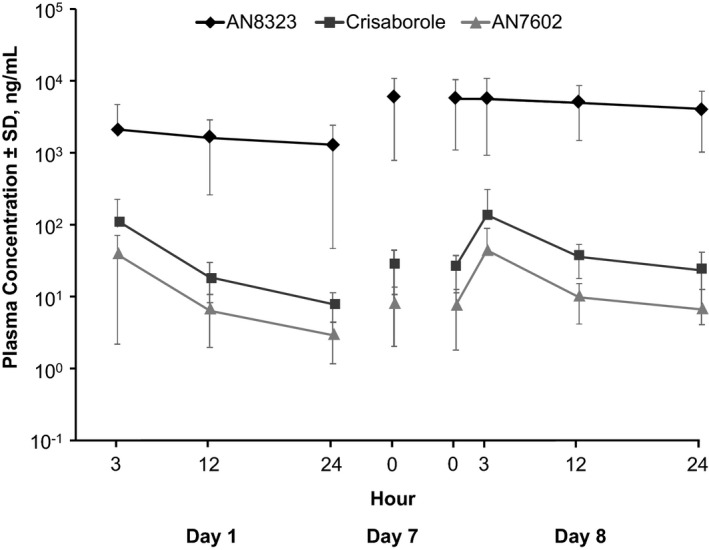
Plasma concentration‐versus‐time curves for crisaborole, AN7602, and AN8323. SD, standard deviation.

Because no significant differences were observed in PK parameters of *C*_max_ and AUC_0–24_ between the three patient cohorts (all p > 0.05), overall PK parameters were calculated based on the full study population. Mean plasma concentration‐versus‐time curves for crisaborole and its inactive metabolites are shown in Fig. 2. Day 1 and day 8 plasma PK parameters for crisaborole and its inactive metabolites (AN7602 and AN8323) are summarized in Table 3.

**Table 3:** Summary of Plasma Pharmacokinetic Parameters for Crisaborole and Metabolites AN7602 and AN8323 on Days 1 and 8 for All Patients

Parameter	Crisaborole	AN7602	AN8323
Day 1			
*T*_max_, hours, median (range) (*n* = 34)	3.00 (3–12)	3.00 (3–12)	12.0 (3–24)
*C*_max_, ng/mL, mean ± SD (*n* = 34)	111 ± 113	37.8 ± 35.0	2,270 ± 2,640
AUC_0–12_, ng·hour/mL, mean ± SD (*n* = 32)	759 ± 730	247 ± 224	16,800 ± 16,900
AUC_0–T_, ng·hour/mL, mean ± SD (*n* = 34)	863 ± 759	288 ± 237	30,800 ± 30,500
AUC_0–24_, ng·hour/mL, mean ± SD (*n* = 29)	833 ± 694	274 ± 207	32,500 ± 32,500
Day 8			
*T*_max_, hours, median (range) (*n* = 33)	3.00 (3–24)	3.00 (0–12)	3.00 (0–24)
*C*_max_, ng/mL, mean ± SD (*n* = 33)	127 ± 196	40.8 ± 48.6	6,150 ± 4,790
AUC_0–12_, ng·hour/mL, mean ± SD (*n* = 32)	949 ± 1240	290 ± 313	63,400 ± 49,000
AUC_0–T_, ng·hour/mL, mean ± SD (*n* = 33)	1320 ± 1310	398 ± 347	119,000 ± 87,600
AUC_0–24_, ng·hour/mL, mean ± SD (*n* = 32)	1320 ± 1330	391 ± 351	116,000 ± 86,700

AUC_0–12_, area under the plasma concentration‐versus‐time curve from time 0 to 12 hours after dosing; AUC_0–24_, area under the plasma concentration‐versus‐time curve from time 0 to 24 hours after dosing; AUC_0‐T_, area under the plasma concentration‐versus‐time curve from time 0 to the last measurable concentration; *C*_max_, observed maximum plasma concentration after dosing; SD, standard deviation; *T*_max_, time to reach *C*_max_.

Crisaborole was rapidly absorbed, with a median *T*_max_ of 3.00 hours on day 1 (range 3–12 hours) and day 8 (range 3–24 hours). Systemic exposure to crisaborole and AN7602 was minimal and increased as the area of application (treatable percentage of BSA) increased. Over 8 days of dosing, there was minimal systemic accumulation of crisaborole and AN7602 in plasma. From day 1 to day 7, AN8323 demonstrated an approximate 3‐ to 4‐fold systemic accumulation in plasma (based on *C*_max_ and AUC from time 0 to 12 hours after dosing [AUC_0–12_]), which remained stable at all day 8 time points assessed. Steady‐state plasma levels of crisaborole and its inactive metabolites (AN7602 and AN8323) were achieved during the dosing phase.

### Safety Results

In the safety population, the mean number of applications ± SD of crisaborole topical ointment, 2% was 53 ± 7 and the mean amount of drug applied was 761 ± 354 g. The mean number of applications of crisaborole was 54 ± 4 in cohort 1, 54 ± 2 in cohort 2, and 51 ± 12 in cohort 3; the mean amount of drug applied was 899 ± 319 g in cohort 1, 834 ± 370 g in cohort 2, and 507 ± 253 g in cohort 3.

Five of 12 (42%) patients in cohort 1, 9 of 12 (75%) in cohort 2, 9 of 10 (90%) in cohort 3, and 23 of 34 (68%) in the safety population reported one or more TEAEs. Nearly all TEAEs (60/63 [95%]) were mild or moderate in intensity and resolved spontaneously, and 36 of 63 (57%) were considered related to study drug. There were no SAEs, and no deaths occurred during the study. One patient, a 2‐year‐old girl in cohort 3, experienced a parent‐reported TEAE of intermittent application site pain and burning that resulted in discontinuation from the study on day 13 at the request of the parent. The most frequently reported TEAEs were application site pain (*n* = 12), AD (*n* = 7), upper respiratory infection (*n* = 3), and application site paresthesia (*n* = 2). The most frequently reported treatment‐related TEAEs were application site pain (*n* = 12), AD (*n* = 4), and application site paresthesia (*n* = 2). No consistent changes in laboratory values, vital signs, or physical examinations were observed.

### Efficacy Results

**Figure 3:**
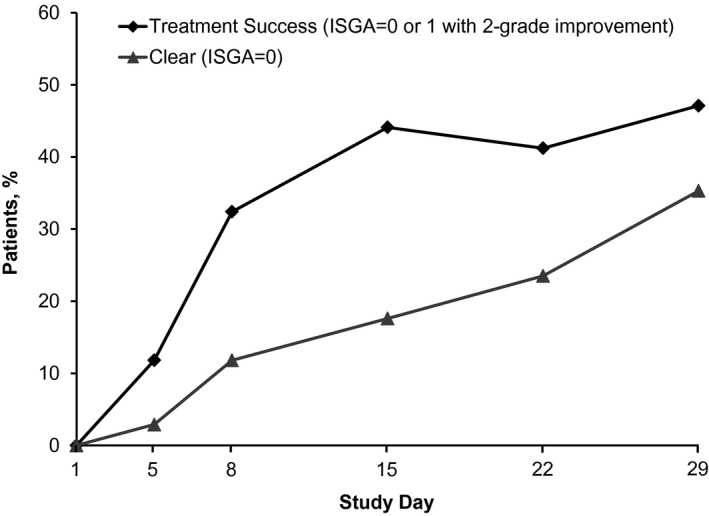
Proportion of patients achieving treatment success or Investigator Static Global Assessment (ISGA) score of clear (0).

Across the study populations, mean ISGA scores declined from 2.65 ± 0.49 at baseline to 1.15 ± 1.08 at day 29. At day 29, 16 of 34 patients (47.1%) achieved treatment success (ISGA score ≤1 with a two‐grade or more improvement from baseline); 19 of 34 (55.9%) achieved treatment success at one or more assessments during the study. At day 29, 22 of 34 patients (64.7%) achieved ISGA scores of 0 (clear) or 1 (almost clear), and the proportion of patients who achieved ISGA scores of 0 or 1 increased steadily throughout the study (Fig. 3). Mean ISGA scores at day 29 and percentage reduction from baseline were 0.58 ± 0.79 (76.4% reduction) in cohort 1, 1.33 ± 1.16 (48.6% reduction) in cohort 2, and 1.60 ± 1.08 (45.0% reduction) in cohort 3.

**Figure 4:**
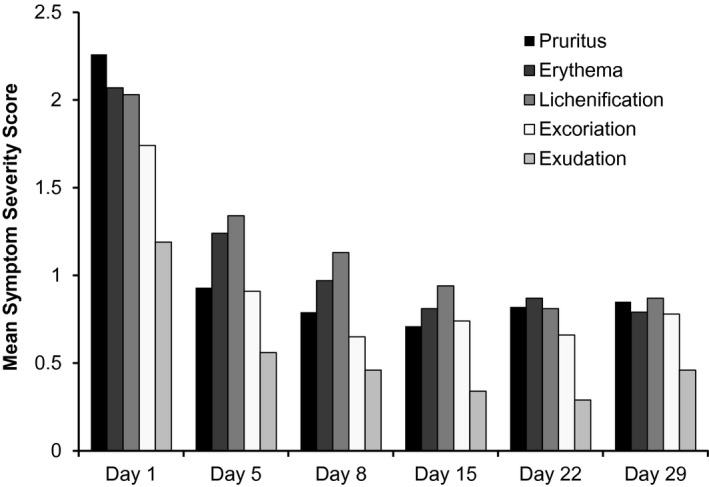
Decrease in mean severity of signs and symptoms of atopic dermatitis.

Mean severity scores for the five AD signs and symptoms from baseline to day 29 are shown in Fig. 4. Improvement in pruritus and all other AD signs and symptoms was observed early in treatment, with the greatest improvement seen between baseline and day 5. Across the full study population, the mean percentage changes from baseline to day 29 in symptom severity scores were erythema, −64.9%; excoriation, −58.2%; exudation, −64.3%; lichenification, −61.3%; and pruritus, −63.3%.

The mean treatable percentage of BSA decreased throughout the study in the overall study population and in each cohort; these results are summarized in Table 4.

**Table 4:** Change from Baseline in Treatable Percentage of Body Surface Area (BSA) After 4 Wks of Treatment with Crisaborole

	Cohort 1, ages 12–17 yrs (*n* = 12)	Cohort 2, ages 6–11 yrs (*n* = 12)	Cohort 3, ages 2–5 yrs (*n* = 10)	Total (*N* = 34)
	Mean ± SD (range)			
Baseline treatable percentage of BSA	35.8 ± 11.3 (27–61)	54.9 ± 19.3 (35–92)	56.9 ± 23.0 (35–91)	48.7 ± 20.2 (27–92)
Day 29 treatable percentage of BSA	5.7 ± 8.6 (0–29)	16.3 ± 14.5 (0–40)	18.6 ± 18.1 (0–46)	13.2 ± 14.7 (0–46)
Percentage change from baseline	−86.6 ± 18.6	−72.8 ± 24.4	−73.0 ± 21.7	−77.7 ± 22.1

## Discussion

Currently approved topical therapies have demonstrated efficacy in relieving signs and symptoms in children and adults with mild to moderate AD 2, 10, but topical corticosteroids and calcineurin inhibitors can be associated with safety risks that may cause patient and caregiver concerns with long‐term use 2, 11. Specifically, long‐term, uninterrupted use of glucocorticoids can cause striae, increase the risk of adrenal suppression, and slow linear growth 12, 13. Calcineurin inhibitors are associated with the potential risk of malignancy (skin cancer, lymphoma), although a direct causal relationship has not been established 2. A novel approach to the treatment of AD involves the inhibition of PDE4, an enzyme that degrades the intracellular second messenger cAMP and initiates the inflammatory cascade; cAMP‐dependent inhibition of PDE4 suppresses this inflammatory response 5, 6. Crisaborole is a topical nonsteroidal antiinflammatory PDE4 inhibitor that has completed phase 3 clinical studies for the treatment of children, adolescents, and adults with mild to moderate AD 8, 9. The chemical structure of crisaborole, a low molecular weight boron‐based small molecule 14, facilitates effective penetration through human skin. Incorporation of boron allows access to target cells in which crisaborole inhibits PDE4. Crisaborole reduces skin inflammation by inhibiting the release of inflammatory cytokines; this occurs through inhibition of PDE4, cAMP‐dependent activation of protein kinase A, and inhibition of the downstream nuclear factor of activated T‐cells and nuclear factor kappa‐light‐chain‐enhancer of activated B‐cell signaling pathways 5, 14.

In this phase 1b maximal‐use systemic exposure study, treatment with crisaborole topical ointment, 2% over 8 days under maximal‐use conditions resulted in minimal systemic exposure of crisaborole and its metabolite AN7602 in children and adolescents with mild to moderate AD. The mean *C*_max_ for crisaborole at day 8 was 127 ng/mL, and steady‐state levels were achieved rapidly after initiation of treatment. There was considerable similarity in PK profiles, with no significant between‐group differences in age‐based cohorts. The crisaborole metabolite AN8323 achieved higher plasma levels and demonstrated 3‐ to 4‐fold accumulation over 8 days. Steady state for crisaborole and its metabolites was reached by day 8. The observed plasma levels are consistent with those previously reported in adults, with adjustments for affected BSA 15.

Crisaborole topical ointment, 2% was generally well tolerated; the most frequently reported treatment‐related TEAEs were application site reactions, which occurred in 12 of 34 patients and were typically transient and self‐resolving. These reactions were mild or moderate in intensity, were not associated with any specific age or cohort, and were not unexpected based on the nature of AD lesions. Only one patient discontinued the study because of a treatment‐related TEAE of application site pain. No SAEs were reported.

Regarding secondary endpoints of efficacy, crisaborole improved disease severity, pruritus intensity, and all other assessed AD signs and symptoms. These findings indicate that crisaborole not only improved the signs and symptoms of AD, but may also have targeted the underlying pathogenesis of the disease. Improvement in these symptoms was observed early after initiation of treatment, with most improvements observed by day 5 (signs and symptoms assessment) or day 8 (ISGA). In addition, crisaborole treatment reduced (by ~75%) the mean percentage of skin with AD lesions.

Limitations of the study included the small number of patients in each cohort, which may limit interpretation of the results. In addition, because the study was designed primarily to assess safety and PK under maximal‐use conditions with open‐label treatment, it lacked an ointment vehicle control arm.

## Conclusions

These results demonstrate that crisaborole topical ointment, 2% appears to be well tolerated and effective, with limited systemic exposure to crisaborole under maximal‐use conditions when administered to children and adolescents with AD involving 25% or more BSA. These findings may support favorable safety and tolerability of crisaborole in children as young as 2 years of age. Phase 2 clinical studies in children and adults with AD have been completed and crisaborole has advanced into phase 3 clinical development for the treatment of mild to moderate AD in patients 2 years of age and older.

## Conflict of Interest

Lee T. Zane, Sanjay Chanda, and Merrie Van Syoc are employees of Anacor Pharmaceuticals. Zoe Diana Draelos, Robert Call, and Eduardo Tschen are clinical investigators for Anacor Pharmaceuticals. Leon Kircik is a consultant and clinical investigator for Anacor Pharmaceuticals. Adelaide A. Hebert is a consultant and clinical investigator for Anacor Pharmaceuticals; all research funding was paid to her employer, the University of Texas Medical School, Houston, Texas.

The authors are fully responsible for the content, editorial decisions, and opinions expressed in this article. Neither honoraria nor other forms of payment was made for authorship.

## Statement of Informed Consent

The study was conducted in accordance with Good Clinical Practice guidelines and the US Code of Federal Regulations and pursuant to the Declaration of Helsinki. All patients (if appropriate) provided assent and their parents/guardians provided informed written consent prior to enrollment.
